# Fifty years of computer analysis in chest imaging: rule-based, machine learning, deep learning

**DOI:** 10.1007/s12194-017-0394-5

**Published:** 2017-02-16

**Authors:** Bram van Ginneken

**Affiliations:** Diagnostic Image Analysis Group, Radboud University Medical Center, Nijmegen, The Netherlands

**Keywords:** Pulmonary image analysis, Computer-aided detection, Computer-aided diagnosis, Image processing, Machine learning, Deep learning

## Abstract

Half a century ago, the term “computer-aided diagnosis” (CAD) was introduced in the scientific literature. Pulmonary imaging, with chest radiography and computed tomography, has always been one of the focus areas in this field. In this study, I describe how machine learning became the dominant technology for tackling CAD in the lungs, generally producing better results than do classical rule-based approaches, and how the field is now rapidly changing: in the last few years, we have seen how even better results can be obtained with deep learning. The key differences among rule-based processing, machine learning, and deep learning are summarized and illustrated for various applications of CAD in the chest.

## Introduction

Gwilym S. Lodwick, a medical doctor from Iowa, first introduced the term computer-aided diagnosis in the scientific literature in 1966, half a century ago [1]. He emphasized that “there is scarcely any repetitive function in which the computer cannot be of help to us, in radiology.” His focus was on the analysis of chest radiographs, about which he published a paper in the journal Radiology in 1963 [2]. He developed a system for predicting from a chest examination—a posterior–anterior and a lateral chest radiograph—whether a patient diagnosed with lung cancer would still be alive one year later. He described his method as a general approach: “a concept of converting the visual images on roentgenograms into numerical sequences that can be manipulated and evaluated by the digital computer.” Nowadays, we would call these numerical sequences feature vectors and their manipulation by a computer is the process of training a classifier. The trained classifier can evaluate feature vectors extracted from new images at test time.

The actual conversion of images into feature vectors was done by Lodwick himself. As a chest radiologist, he thought up a long list of visually assessable items that he could score on radiographs. He called this a “complete descriptive system”. These items, such as the sharpness of the margin of the tumor in both views, or the size of the cancer, or the presence of cavities, were not assessed by the computer because, in 1963, it was not yet possible to scan a radiograph and process the image in the computer memory. This type of work started in the 1970s. Image processing in those days typically consisted of application of many different low-level operations such as filtering for detecting edges and lines, extraction of regions by connecting pixels with similar characteristics (region growing), and fitting of simple mathematical structures, such as lines, circles, and ellipses, e.g., with a Hough transform, to the data.

In the 1970s, the two-stage concept that Lodwick had proposed (converting the images to numerical sequences, manipulating the sequences) was usually not followed. Instead, longer algorithms in which these low-level image processing operations were concatenated were proposed to perform a comprehensive analysis of a scan. A good example is the work of Toriwaki et al. [3]. This study describes step-by-step procedures for finding in chest radiographs the lungs, the heart, the ribs, and finally abnormal regions. This approach is what I will refer to as *rule*-*based* in this study. There is a clear analogy with the expert systems with many if–then-else statements that were popular in artificial intelligence in the 1970s. These expert systems have been described as GOFAI (good old-fashioned artificial intelligence) and were often found to be brittle, similar to rule-based image processing systems.

Computer-aided diagnosis (CAD), with the two-step approach advocated by Lodwick, became more popular in the 1980s and beyond, and it was widely applied to chest imaging in the seminal work of the group of Kunio Doi at the University of Chicago [4]. In CAD, the image analysis problem is translated into a pattern recognition or machine learning problem (in this work I use the latter term, but both terms could be used, good textbooks on the subject are [5, 6]) in which features are extracted from complete image or, more typically, regions in the image, and a computer is trained to classify feature vectors.

Until recently, most CAD practitioners would have expected that this would remain the dominant approach to automated image analysis. However, the process of deciding which are the optimal features for solving a particular problem at hand is very complex. It is generally impossible to prove that a set of features is optimal; choosing a set of features is, in a way, more art than science. In the step from completely rule-based approaches to machine learning, the task of optimally extracting information from the feature vectors was taken from the human who designed the system to the computer, because a computer is better able to construct a decision function from large amounts of information. Taking this perspective, one wonders whether the process of converting images into features could also not be done better by computers.

This is where deep learning comes in, and takes over from the traditional machine learning approach where human experts define the set of features to be extracted from images. In deep learning, a network takes images, or regions in images, as input and transforms these, via many layers of processing steps, into a decision. In these intermediate layers, the feature extraction takes place, and these features are not explicitly constructed by the designers of the system, but are learned from the data during the training process. This is a complete paradigm change that has been called by some the end of code.^1^

In this study, my goal is not to give a complete overview of computer analysis of chest radiographs and computed tomography images. I have previously reviewed CAD in chest radiography [7] and computed tomography [8], and more recently I surveyed chest X-ray applications [9] and segmentation in chest CT [10] and discussed how to move CAD to the clinic [11]. Instead, this study will illustrate how these three approaches—rule-based image processing, with machine learning, and with deep learning—have been applied to several important problems in chest image analysis, and how deep learning is currently becoming the dominant approach with very promising results.

The next section provides a brief introduction to image analysis with deep learning. I then discuss one application in chest radiography analysis and four in chest CT. “Section 8” is the conclusion.

## Deep learning in image analysis

Deep learning uses models (networks) composed of many layers that transform input data (i.e., the images) to outputs (e.g., disease present/absent, or pixel/voxel belonging to object/background). The most successful type of models for image analysis to date, and the only one I will discuss in this work, are convolutional networks (convnets), which contain many layers that transform their input with convolution filters that typically have only a small extent.

Work on convnets dates back to the 1970s [12], and already in 1995, they were applied to medical image analysis by Lo et al. [13]. The work of Suzuki et al. discussed below also directly processed image patches with a neural network in a variety of medical image analysis tasks, but did not employ convolutional layers in the network. The first successful application of convnets, which was also commercialized, was LeNet by Lecun et al. [14]. It used small 32 × 32 gray-scale images of hand-written digits. These images were preprocessed by rule-based image processing to have the right contrast and the digit centered in the image. The network contained three convolutional layers, and, in total, 60,000 parameters that were all learned from the data via backpropagation. This is called end-to-end learning, as all parameters in the entire chain from image to classification output are learned at the same time in a single iterative process.

Despite the success of LeNet, the use of convnets for image analysis did not gather much momentum until 2012. The watershed event was the entry of Krizhevsky et al. [15] to the ImageNet^2^ challenge in December of that year. The proposed deep convolutional network won that competition by a large margin, smashing records from previous years. Their AlexNet contained 60 million parameters—a thousand times the number of LeNet—and performed a 1000 class classification on much larger (224 × 244) color images. The most important reasons why convnets were now able to perform successfully on these much larger problems were: (1) new techniques developed for more efficiently training deep networks; (2) availability of many more training data; (3) advances in parallel computer processing with GPUs. In subsequent years, enormous further progress was made in image classification by use of related but deeper architectures [16]. In computer vision, deep convolutional networks are now the technique of choice for image analysis.

**Fig. 1:**
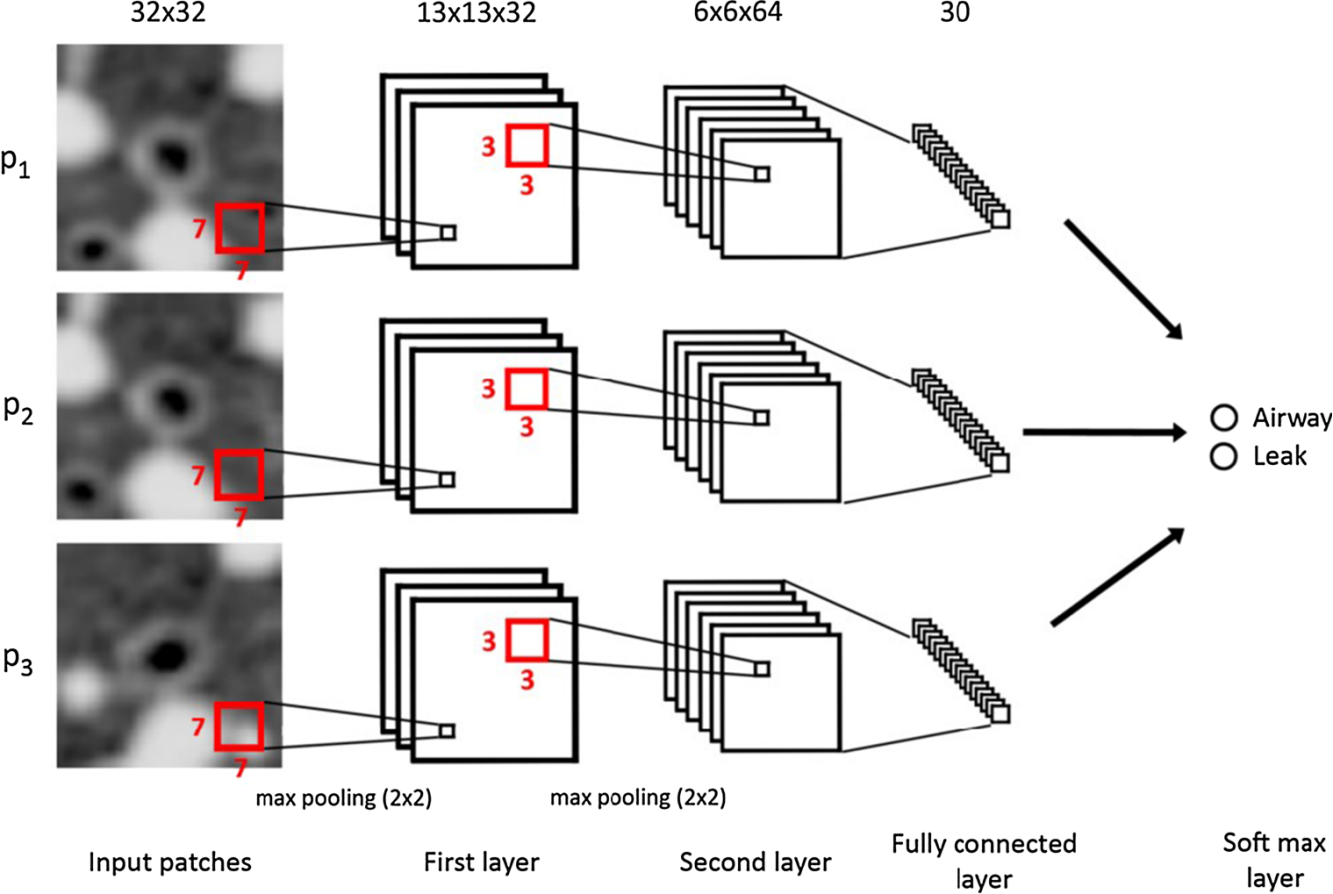
Typical example of a convolutional network. This network was used to analyze three 32 × 32 patches extracted from chest CT scans that can either represent a true airway branch or a leakage. This architecture was used in [20]

For details on convnets and deep learning, see overviews by Schmidhuber [17] and LeCun et al. [18]. A good overview of earlier techniques for learning features (so-called representation learning) can be found in Bengio et al. [19]. Figure 1 provides a basic overview of a convnet that was used in a recent publication on airway extraction from chest CT data [20]. In this example, three patches are processed in parallel. This illustrates the versatility of such networks; they can be put together in many different configurations. Parameters (weights) can be shared across different parts of the network and all learnt directly from the data. In this example, each patch of 32 × 32 pixels is first processed by a set of 32 filters of 7 × 7. Valid convolutions are used; therefore each filtered image has a size of 26 × 26. After the convolutional layer, a non-linear filter is applied (a rectified linear unit, or ReLu for short [21], one of the important algorithmic improvements made to be able to train deep networks better) and the image is subsampled by a factor of 2 with max-pooling (another technique that was not used by LeCun in 1998, but now a standard approach, although better choices may be possible). This leaves us with 32 images of 13 × 13. These are subsequently processed by 64 filters of 3 × 3, again applying ReLu and max-pooling, resulting in images of 6 × 6. The 2304 voxels in these images (6 × 6 × 64) are fully connected to 30 neurons, and the three groups of 30 neurons are concatenated and used as input to the final classification layer.

The proper implementation of software for building and training such networks is far from trivial. An important reason why the techniques have been taken up so quickly is the availability of several open source frameworks available to construct, train, and run these networks, such as Theano, Caffe, Tensorflow, and many packages that have been written on top of these frameworks, such as Lasagne and Keras, to name just a few. A good starting point is http://deeplearning.net/software_links/.

The medical image analysis research community has taken notice of the large successes of convnets in computer vision and in 2015 and 2016 more than 300 papers were published on applications of deep learning in workshops, conferences, journals, and special issues [22].

## Rib detection and suppression in chest radiographs

The detection and suppression of ribs in chest radiographs have received a lot of attention. Toriwaki et al. [3] were among the first to describe rule-based algorithms to detect the ribs. They first estimated the approximate location of rib borders by looking for horizontal lines with a 5 × 1 filter. The output of this filter was thresholded and refined with 11 × 11 filters for the central, middle, and peripheral parts of the ribs. Coefficients in the filters were not learned but hand-picked based on assumptions about the rib border width and orientation. Next, quadratic functions were fitted to the points on the rib borders. Several variations on such approaches were published in later years, and even 25 years later Vogelsang et al. [23] published a similar approach. In addition, Vogelsang et al. [23] attempted to suppress the rib borders by assuming a simple parametric model of the rib border profile, fitting this model to the data at the located rib borders, and subtracting the profile from the images. The authors hypothesized that this suppression could be of help in the further analysis of the images.

Later, supervised methods were introduced for rib cage extraction. van Ginneken and ter Haar Romeny [24] constructed a statistical shape model of the posterior rib borders, trained with 35 images, and fitted this to the data by finding model parameters that generated a rib cage with borders located at positions where the edges pointed from both sides toward the rib border. Loog and van Ginneken [25] computed a set of features based on Gaussian derivatives for every pixel in the lung fields after first locally normalizing the image. After feature extraction and classification, this yields a rough estimate for each pixel to be part of the costal or intercostal space. Subsequently, this pixel output was refined using the output of neighboring pixels as additional contextual features.

Hogeweg et al. [26] combined the approach of van Ginneken and ter Haar Romeny [24] and of Vogelsang et al. [23] by creating statistical models with principal component analysis for the profiles along the rib borders. Fitting these profile models to the data and subtracting them resulted in reasonably convincing rib suppression, and the same suppression mechanism was later shown to be capable of removing other elongated structures (clavicle shadows and catheters) from chest radiographs as well [27].

An important step toward the philosophy of deep learning was made by Suzuki et al. [28]. In this work, they processed 9 × 9 pixel patches in chest radiographs, directly estimating with the 81 raw pixel values as input, the value of the central pixel in a bone image from dual-energy images. The estimation process was done by a neural network with one fully connected intermediate layer (no convolutional layers were used). Subtracting the estimated bone images yields a virtual soft tissue image in which rib borders are suppressed. Suzuki et al. [28] use a multi-resolution decomposition of the image to perform the suppression at multiple scales, which led to better results. Suzuki has used his patch-based neural network approach for many other tasks in 2D and 3D medical image analysis, notably nodule detection in chest radiographs and chest CT [29, 30].

The same task of estimating bone images and soft tissue images for a given radiograph, trained with dual-energy radiographs, was addressed by Loog et al. [31]. In this work, the set of input features did not consist of raw pixel values but of a set of Gaussian derivatives. This work can also be seen as an attempt to learn a complex non-linear filter directly from the pixel data; hence, the phrase ‘filter learning’ in the title of their article.

Recently, Yang et al. [32] presented a cascade of convolutional networks with three convolutional layers, trained with 404 dual-energy chest exams to estimate, and subtract, the bony image from the input image to obtain a virtual soft tissue image. The authors use a multi-scale approach and estimate the gradient of the bone images successively from coarse to fine scales. The authors show that using a large number of filters leads to improved results. The soft tissue images produced are visually highly convincing, and the technique can also be applied to radiographs from different sources.

This summary of more than 40 years of research shows how rule-based schemes were used initially for finding ribs and producing very coarse rib suppression. Machine learning and statistical modeling, trained with more data, improved the quality of rib detection and suppression. The recent application of deep learning to the problem of rib suppression shows great potential and represents a major step forward in the learning of complex filtering applications which have many possible applications in medical imaging.

## Fissure extraction from CT

Pulmonary fissures are the boundaries of the lobes of the lungs. They consist of a double layer of visceral pleura and are visible as lines on CT and as sheets in 3D. It is relevant to locate the fissures for many reasons. For example, diseases are often contained within lobes, and spreading across a fissural boundary should be noted. Nodules can be attached to fissures, and if they have a triangular shape, they are very unlikely to represent a malignancy [33]. New bronchoscopic treatments for severe COPD can be applied only if a diseased lobe has a complete fissure along its boundary [34].

The work of van Rikxoort et al. [35] directly compares a rule-based approach to fissure extraction with a machine learning approach. The rule-based approach was previously proposed by Wiemker et al. [36] who reasoned that the Hessian matrix of second order derivatives can be used for deducing whether a voxel is likely to be on a sheet-like bright structures. They computed for each location the three eigenvectors of the Hessian matrix, sorted by absolute size, |*λ*_0_| ≥ |*λ*_1_| ≥ |*λ*_2_|. For a voxel located on a fissure *λ*_0_, the second derivative in the direction for which it is largest, should be high, because along this direction one travels from the lung parenchyma, through the fissure, into the lung parenchyma again. In the two other directions, a small eigenvalue is expected, as one moves along a locally flat structure with a constant intensity. Wiemker et al. [36] derived a formula for enhancing sheets, and they add a term that selects for voxels with an intensity similar to a fissure. The analysis can be done at multiple scales, and the largest output across scales is taken. This approach is very elegant, and similar filters have been constructed for enhancement of nodules (three large positive eigenvalues), vessels (two large and one small eigenvalue), and more complex structures such as vessel bifurcations [37].

van Rikxoort et al. [35] compared this approach with a voxel classifier that takes a number of Gaussian derivatives, 57 in total, for each voxel, and classifies the likelihood of the voxel to be on a fissure using feature selection and a *k*-nearest-neighbor classifier. The authors note that in the resulting voxel probability map, fissures are again visible as plates, and they repeat the process using the probability map as input in order to suppress spurious responses. The results of the study convincingly demonstrate that the machine learning approach is superior to the rule-based filter. The latter especially has difficulty with noisy lower dose scans where the reasoning that led to the analytical form of the filter is apparently not entirely valid.

The message here is that it can be better to learn a complex filter from the data, instead of attempting to derive it using intuitive reasoning and modeling. The approach of learning filters or creating voxel classifiers has been the topic of many studies. One of the first studies is the work of Ochs et al. [38], who detected airways, fissures, nodules, vessels, and lung parenchyma using voxel classification in chest CT.

## Airway segmentation in CT

The extraction of airways from CT scans is important for a variety of applications: measurements of airway lumen size and wall thickness are predictive of obstructive lung diseases such as COPD, and they are directly affected in diseases like bronchiectasis; airway segmentation can be used for planning of procedures such as bronchoscopy, and knowledge about the precise locations of airways can be used for improving the segmentation of other structures and the detection of abnormalities such as endobronchial nodules.

Airway extraction from CT is a topic that is highly amenable to rule-based processing. The prototypical method would start by locating a seed point in the trachea and from there connecting voxels with air density, close to –1000 Hounsfield units (HU) to the seed. As the airways are surrounded by airway walls with tissue density (around 0 HU), this approach should in theory extract the full airway tree. In practice, that does not work because of noise, the fact that lung parenchyma consists around 90% of air and in case of emphysema may have values close to that of the airways, and partial volume effects. Growing the airways using only density will therefore “leak” into the parenchyma. A variety of rules can be constructed for detecting and preventing leakage. Our approach [39], that was inspired by Schlathölter et al. [40] and Kiraly et al. [41], used 5 sets of rules to prevent leakage while still growing the tree as much as possible. The method worked well, extracting several meters of airway and hundreds of branches far into the periphery of the lung for some scans, but it did not even extract the tree up to a segmental level in others.

In 2009, we carried out a large comparative study for airway segmentation, called EXACT’09 [42]. Fifteen teams participated with a method to segment 20 test scans. All methods were evaluated with exactly the same protocol. All airway branches detected by any method were visually inspected by trained human observers who used various reconstructions and visualizations. Every branch was either accepted as a valid airway or rejected because it contained non-airway voxels.

All methods except for one were rule-based. The exception was a method described by Lo et al. [43]. The backbone of this machine learning-based approach, which is coined an airway appearance model, is a voxel classifier (the authors use a *k*-nearest-neighbor classifier) that differentiates between airway and non-airway voxels. The authors wrote: “This is in contrast to previous works that use either intensity alone or hand crafted models of airway appearance.” They refer to Ochs et al. [38] who introduced the concept of voxel classification in chest CT, as we already mentioned above. The output of this voxel classifier is post-processed with a scheme that is similar to other rule-based airway extraction methods, but the authors claim that “applying the region growing algorithm on the airway appearance model produces more complete airway segmentations, leading to on average 20% longer trees, and 50% less leakage.”

Recently, the first method that employs deep learning for airway extraction has been published [20]. Like that of Lo et al. [43], this method is not a completely new approach but builds upon classical rule-based approaches. Any existing method or methods can be used as a basis. The authors observe that existing rule-based schemes typically have a variety of free parameters that can be adjusted. For one particular test scan, running a method with many different settings will be in total extract many more airways than with just a single (optimal) setting. But these extra detections come at the expense of many additional false positive detections (leakages) as well. This study is where the authors resort to deep learning with a convolutional network. They inspect every branch in the union of many rule-based segmentations obtained with different settings. They extract three image patches of 15 × 15 mm and 32 × 32 pixels that are processed by two convolutional layers of filters of 7 × 7 and 3 × 3 and max-pooling layers, followed by a fully connected layer. The network is used for finding leaks and pruning the segmentation to remove them. If this procedure breaks the connectivity of the airway tree, disconnected branches are reconnected. The results are evaluated on the EXACT’09 data set and outperform the other airway segmentation methods in that challenge.

## Nodule detection in CT

Pulmonary nodules may represent lung cancer. The key to detecting nodules in chest CT scans is differentiating them from vessels. Both nodules and vessels have tissue density, surrounded by much lower parenchyma density; nodules are spherical and vessels are cylindrical. These observations can be used for construction of a rule-based scheme for differentiating nodules from vessels. The first system to do this in 3D was proposed by Wiemker et al. [44]. The scheme worked remarkably well, with a reported 95% sensitivity at 4.4 false positives per scan. The test set, however, was limited to 12 cases with no less than 203 nodules. These cases contained a large number of lung metastases which are known to be smooth and highly spherical.

Many authors proposed systems that followed the standard machine learning approach for nodule detection. The earliest systems were 2D, because only in the early 2000s it become routine to obtain isotropic CT scans of the lungs, allowing for 3D analysis. My group [45] developed a 3D approach consisting of candidate detection based on finding clusters of voxels with an appropriate isophote curvature and shape index, computing 18 features and a first classifier to reduce false positives, and computing another 135 features and reclassifying the remaining candidates.

In 2009, I organized a comparative study called Automated NOdule Detection (ANODE09^3^) [46]. The study had a test set of 50 CT scans containing 207 nodules, and results for 12 systems were submitted. The best systems achieved a sensitivity of 70 to 75% at 4 false positives per scan. This is in line with results reported in the literature for other machine learning-based systems applied to other databases. A commercialized version of the rule-based system of Wiemker et al. [44] did poorly on ANODE09, but interestingly, when combined with other systems, it tended to boost the results substantially, indicating that this rule-based approach was complementary to the feature-based systems.

A drawback of the ANODE09 dataset was that it originated from a single center and contained mostly small nodules which have a very low likelihood of representing cancer. In 2016, my group therefore again prepared a nodule detection challenge called LUNA16.^4^ The dataset was collected from what is currently the largest publicly available reference database for lung nodules: the LIDC-IDRI set [47], available from the NCI Cancer Imaging Archive.^5^ The LIDC-IDRI database contains a total of 1018 CT scans. The database is heterogeneous, consisting of clinical dose and low-dose CT scans collected from seven academic institutions, and a wide range of scanner models and acquisition parameters. LUNA16 used 888 scans (LIDC-IDRI scans with thick slices and DICOM errors were discarded). My group used the same dataset for our convolutional network based nodule detection system [48], and we were curious to learn from experiences of other groups working with the same data.

LIDC data have been used by many groups, including Wiemker’s group which recently published a machine learning-based nodule detection system that uses the LIDC database [49]. With LUNA16, systems can be compared for the first time on the same subset of LIDC data, with the same evaluation protocol. The results, recently summarized by Setio et al. [50], are unambiguous: systems based on convnets perform substantially better than do classical machine learning approaches. LUNA16 has two tracks: a track for complete systems and a track where systems process a set of nodule candidate locations. These candidates are computed by merging of the output from five different rule-based algorithms for finding nodule candidates. At the time of this writing, the best results are obtained by systems that use these candidates, but systems that rely completely on convnets in their entire processing chain are already almost as accurate, achieving around 90% accuracy with 1 false positive detection per scan.

## Nodule classification and characterization in CT

In nodule classification and characterization, we observe the same trend as in nodule detection: recent systems use deep learning to infer the type of nodule or an estimate of malignancy.

Until recently, only machine learning approaches were used for this task. One of the first studies to estimate malignancy was presented by McNitt-Gray et al. [51], who analyzed a dataset of 14 benign and 17 malignant nodules in a leave-one-out approach and a linear discriminant classifier with feature selection. A set of well over one hundred 2D features based on the size, density, shape, and texture of the nodules was computed. Additional systems are reviewed by Suzuki [52]; all use standard features, some 3D, and classifiers such as linear classifiers and support vector machines. An exception is the work of Suzuki et al. [53], who presented a scheme directly using the pixel data from patches extracted around the nodules to estimate the probability of malignancy with a fully connected neural network.

The medical literature also proposes systems for inferring the probability of malignancy for a nodule using logistic regression on a small set of sensible features such as nodule size (the most important factor if only a single scan is available and the growth rate cannot be assessed), type (sold, part-solid, non-solid), location (upper lobe or not), spiculation (yes/no), other signs from the CT scan such as the number of nodules and the presence of emphysema, and clinical information about the patient. The best known model is the PanCan model by McWilliams et al. [54], published in the New England Journal of Medicine, which was derived from a Canadian screening program for which 102 cancerous and 6906 benign nodules were available. The model was validated on a different Canadian screening cohort.

Recently, Ciompi et al. [55] presented a method for inferring the nodule type with use of a convolutional network. Together with automated nodule detection (discussed above), rule-based nodule segmentation [56, 57], robust emphysema quantification [58], and lobe segmentation [59], all elements are in place for automatically performing PanCan malignancy probability assessment for all nodules in a scan. Of course, a step further would be to forget about a model based on a small set of classical features and use deep learning directly to estimate the probability of malignancy, such as was done, e.g., by Shen et al. [60]. The model in that work, however, was trained with radiologists’ estimates of nodule malignancy probability, which is a major limitation. Also, ideally one would like to analyze scans of a nodule obtained at multiple time points, as information about growth is known to be the most important cue for malignancy.

In January 2017, the data scientist community Kaggle, has started a competition^6^ with $1 million in prize money to estimate the probability that a person was diagnosed with lung cancer within one year after a chest CT, available to the participants, was obtained. In the Data Science Bowl in 2016 and 2015, on cardiac MRI analysis and detection of diabetic retinopathy from fundus photographs, respectively, all leading solutions were based on deep learning. This is likely to be the case for this competition as well.

## Concluding remarks

**Fig. 2:**
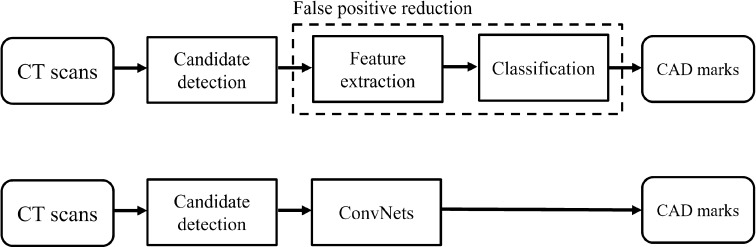
*Top*: setup for a “traditional” CAD system for nodule detection in CT. *Bottom*: plugging in convnets to perform false positive reduction

As illustrated by the five applications that I discussed in the preceding sections, the field of computer analysis of chest images has seen a transition from developing purely rule-based systems to using training data and extracting features from images and processing these with various classifiers. Both paradigms are typically combined: in computer-aided detection systems, rule-based image processing is often used for finding candidates, followed by feature extraction and classification for each candidate. Recently, the research community has embraced deep learning, in particular convolutional networks. One way of looking at this development is to consider convnets simply as a new way of feature extraction, which can be “plugged in” at the appropriate place in an existing processing pipeline. More precisely, convnets function as feature extractors and classifiers in one. This is illustrated in Fig. 2 for the example of nodule detection in CT (but it would be similar for most CAD applications): convnets replace to so-called false positive reduction step. Solutions submitted to LUNA16, however, indicate that it is certainly possible to obtain good results using one convnet, or two convnets in succession, for both the candidate extraction and the false positive reduction step.

Examples from this survey study show that convnets can be used in other ways as well, to produce filtered images, i.e., chest radiographs without rib shadows, and to remove leaks produced by an aggressive traditional airway extraction algorithm.

The potential advantages of convnets are not merely that they are better feature extractors. Their general applicability should make it possible to develop new applications much more quickly. Recent results from the ImageNet challenge show that a single deep convolutional network can recognize 1000 different objects with an accuracy comparable to that of humans. This indicates it may be possible to make computer-aided detection systems that can simultaneously locate many different types of abnormalities in particular scans.

The fact that deep learning is also an excellent technology for text analysis allows one to combine analysis of radiology text reports with medical image analysis. The work of Shin et al. [61] and Wang et al. [62] is a first step in this direction. The authors employ text analysis and generate captions for chest radiographs automatically.

I expect to see more results in this direction in the next ten years, and automated reporting for chest imaging may become a reality.

## Footnotes

1
https://www.wired.com/2016/05/the-end-of-code/.

2
http://image-net.org/.

3
https://anode09.grand-challenge.org/.

4
https://luna16.grand-challenge.org/.

5
https://wiki.cancerimagingarchive.net/display/Public/LIDC-IDRI.

6
https://www.kaggle.com/c/data-science-bowl-2017.
